# Analysis of standard vs dose-escalated stereotactic body radiation therapy in localized prostate cancer: a comparative evaluation of survival outcomes

**DOI:** 10.3389/fimmu.2025.1654174

**Published:** 2025-08-13

**Authors:** Bichun Xu, Xianzhi Zhao, Tingting Wei, Yiyin Liang, Weiwei Zhang, Liang Chen, Zuping Lian, Huojun Zhang

**Affiliations:** ^1^ Department of Radiation Oncology, The First Affiliated Hospital of Naval Medical University, Shanghai, China; ^2^ Department of Radiotherapy, Tongji Hospital, School of Medicine, Tongji University, Shanghai, China; ^3^ Department of Radiotherapy and Oncology, the Second Affiliated Hospital of Soochow University, Suzhou, China; ^4^ Department of Medical Oncology, Ruikang Hospital Affiliated to Guangxi University of Chinese Medicine, Nanning, China

**Keywords:** stereotactic body radiation therapy (SBRT), prostate cancer, localized, dose-escalated, overall survival

## Abstract

**Background and purpose:**

This study aimed to compare the safety and efficacy of high-dose biologically effective dose (BED) versus standard dose regimens in stereotactic body radiotherapy (SBRT) for localized prostate cancer (PCa) using a propensity score matching (PSM) analysis.

**Methods:**

Between June 2012 and February 2022, prostate-localized SBRT patients from two institutions were retrospectively reviewed. The high-dose group (n=12) received high-dose BED_1.5_ (>250Gy), and the control group (n=119) according to NCCN guidelines (35-37.5 Gy/5f, BED_1.5_ 198.3-225Gy). PSM was performed in a 1:4 ratio based on key clinical variables. Survival outcomes, including overall survival(OS), cancer-specific survival (CSS), biochemical progression-free survival (bPFS), local control (LC), and distant metastasis-free survival (DMFS)were analyzed using Kaplan-Meier methods with SPSS v26.

**Results:**

In the 7-year follow-up, the high-dose group exhibited a 66.7% OS rate vs. 83.4% in controls (p=0.402) and an 88.9% CSS rate compared to 90.5% in controls (p=0.480). The high-dose group demonstrated a 91.7% 7-year bPFS rate, while controls had a 67.4% rate (p=0.497). Higher gleason score correlated with impaired biochemical control (p=0.028), and adverse NCCN classifications indicated suboptimal control (p=0.028). The high-dose group achieved a 100% 7-year LC rate vs. 95.1% in controls (p=0.569) and a 91.7% 7-year DMFS rate compared to 81.6% in controls (p=0.918). Patients with pre-existing health conditions were less likely to develop distant metastasis (p=0.047). Most patients tolerated SBRT with minimal toxicity, and no grade 3 or higher adverse events were observed.

**Conclusion:**

Escalating the biologically effective dose above standard levels did not yield a significant improvement in tumor control or survival outcomes compared to conventional SBRT dosing for localized PCa. Further prospective studies are warranted to clarify the role of dose escalation in this setting.

## Highlights

- Retrospective study of high vs. standard dose BED in SBRT for localized PCa.
- Standard doses remain effective in achieving comparable outcomes to high-dose regimens.
- High BED in SBRT for localized PCa did not significantly enhance tumor control or survival rates.

## Introduction

For localized prostate cancer (PCa), standard treatments include active surveillance (AS), radical prostatectomy (RP), and external beam radiotherapy (EBRT) (1). Nonetheless, these options lead to significant adverse effects, such as urinary incontinence and erectile dysfunction (2). A systematic review of 72 studies, including focal therapy (FT) modalities like focal brachytherapy, shows promising early results, though long-term oncological effectiveness remains uncertain. High-quality evidence suggests minimal adverse reactions with FT (3). Notably, most FT approaches require repeated general anesthesia, impacting patients’ quality of life (QoL). An alternative, stereotactic body radiotherapy (SBRT), offers precise treatment without anesthesia, demonstrating efficacy comparable to intensity-modulated radiation therapy (IMRT) (4).

These evolving strategies illustrate the trend in oncology toward precise, minimally invasive treatments that aim to reduce toxicity while maintaining effectiveness, driven by advances in biology and technology (5). SBRT offers several advantages (6). Firstly, escalating the dose enhances cancer control. Secondly, the low α/β ratio of prostate cancer (estimated at 1-2) leads to significant relative biological effectiveness (RBE) with SBRT, increasing the biologically effective dose (BED) (7). Consequently, this low α/β ratio of prostate cancer renders it particularly sensitive to hypofractionated high-dose radiotherapy. Implementing individualized high-dose fractionation protocols can thus enhance tumor control while simultaneously minimizing the radiation dose to surrounding normal tissues, ultimately reducing the incidence of late radiation-induced toxicity. Lastly, the CyberKnife system provides precise, image-guided radiation delivery, minimizing exposure to critical organs and enhancing patient convenience during extensive radiation procedures.

Hypofractionated radiation therapy, notably SBRT, gains popularity for localized PCa due to its safety and convenience. Trials explore dose escalation benefits in conventional radiation (8, 9), with hypofractionation (42.7 Gy/7f) proving non-inferior for intermediate and high-risk patients (4). The current National Comprehensive Cancer Network (NCCN) guidelines recommends at least 36.25 Gy/5 fractions based on PACE-B research (10, 11). However, earlier NCCN versions have acknowledged that lower SBRT doses, such as 35 Gy/5 fractions, may still be acceptable. Notably, the NCCN also endorses fractionation schemes like 9.5 Gy*4 fractions or 8 Gy*5 fractions, each resulting in a BED greater than 250 Gy, while simultaneously recognizing regimens with BEDs below this threshold. Ongoing debates continue regarding the optimal SBRT dose, with the impact of delivering higher BEDs—such as 40 Gy/5 fractions—on survival outcomes yet to be explored in cohort studies. This retrospective study evaluates the influence of dose escalation on survival outcomes between higher BED (>250) and recommended fractionation doses.

## Materials and methods

### Patient selection

Patients receiving prostate-localized SBRT without regional lymph node involvement or distant metastasis from two research medical institutions (June 2012 - February 2022) were selected. Exclusions included prior radical surgery, brachytherapy, or proton therapy. Inclusion criteria comprised confirmed prostate adenocarcinoma, enhanced pelvic magnetic resonance imaging (MRI), emission computed tomography (ECT), Eastern Cooperative Oncology Group (ECOG) performance score ≤ 1, and those unsuitable for surgery due to medical conditions. Informed consent was obtained from all enrolled patients prior to treatment. Data included age, ECOG status, prostate-specific antigen (PSA), Gleason score grading, clinical T-stage, NCCN risk group, prior surgery/androgen deprivation therapy (ADT), and SBRT details. The high-dose group (n=12) had BED_1.5_ (α/β=1.5Gy) >250Gy, while the controls (n=119) followed NCCN guidelines (35-37.5 Gy/5f, BED_1.5_ 198.3-225Gy). Approved by the institutional review board, this study adhered to the Helsinki Declaration.

### SBRT protocols

Before formulating the radiation therapy plan, two or four fiducial markers were implanted into the prostate. Patients were positioned supine, arms resting at their sides, and secured with a thermoplastic mask. One week post-marker insertion, an enhanced computed tomography (CT) scan (1.5 mm slice thickness, 10 cm above and below the prostate) was conducted. For low-risk prostate cancer, the clinical target volume (CTV) covered the entire prostate. In the intermediate-risk group, CTV included the prostate and a 1-centimeter margin around seminal vesicles. For the high-risk and very high-risk groups, the CTV comprised the entire prostate and a 2-centimeter margin around the seminal vesicles. If the tumor invaded seminal vesicles, CTV covered the entire prostate and seminal vesicles. SBRT was administered using CyberKnife (Accuray Corporation, Sunnyvale, CA, USA).

The planning target volume (PTV) expanded by 5mm in all directions (excluding posterior), and CTV expanded by 3mm to minimize rectal radiation. **Table 1** summarizes treatment parameters for both approaches. The distribution graph of BED_1.5_ for the two patient groups can be found in **Supplementary Figure S1**. The control group received a prescription dose of 35-37.5 Gy in 5 fractions every other day, with the median prescription isodose line at 79%. BED was calculated using the standard linear-quadratic model (α/β = 1.5Gy, common for prostate cancer). Dose-volume constraints for organs at risk (OAR) included rectum (V_18.1 Gy_ < 50%, V_29 Gy_ < 20%, V_36 Gy_ < 1 cc), bladder (V_18.1 Gy_ < 40%, V_37 Gy_ < 10 cc, optimal V_37 Gy_ < 5 cc), prostatic urethra (V_42 Gy_ < 50%), femoral head (V_14.5 Gy_ < 5%), penile bulb (V_29.5 Gy_ < 50%), and intestine (V_18.1 Gy_ < 5 cc, V_30 Gy_ < 1 cc) (12).

**Table 1:** Treatment parameters used for radiotherapy.

Parameters	Total	The high-dose group	The control group
Clinical Target Volume (ml)	55.6 (8.0-182.7)	34.2 (8.0-122.0)	61.0 (23.8-182.7)
Total prescribed dose (Gy)	36.9 (35.0-42.0)	39.6 (36.0-42.0)	36.2 (35.0-37.5)
Number of fractions	5 (3-5)	4 (3-5)	5
Dose per fraction (Gy)	8.1 (7-13.3)	12.3 (8-13.3)	7.2 (7.0 -7.5)
BED_1.5_ (Gy)	236.7 (198.3-395.4)	339.01 (253.3-395.4)	211.0 (198.3-225)
Number of fiducials	4	2	4
Prescription isodose line (%)	76 (57-85)	65 (57-72)	79 (71-85)

All data were shown as median values (range).

BED_1.5_: biologic equivalent dose (α/β=1.5 Gy).

### Response evaluation and follow-up

Post-radiation, monthly assessments monitored PSA and testosterone levels. Biochemical progression was defined as PSA increase ≥ 2 ng/mL from nadir (13). Overall survival (OS) was calculated from radiation therapy start to final follow-up or death. Cancer-specific survival (CSS) was defined as the time to death resulting from prostate cancer progression. Biochemical progression-free survival (bPFS) was from SBRT initiation to biochemical progression or last follow-up. Local control (LC) denoted no progression at the primary site. Distant metastasis-free survival (DMFS) calculated from radiation therapy start to clinical metastasis diagnosis or patient’s death. Acute and late toxicities assessed by Common Terminology Criteria for Adverse Events (CTCAE) v5.0.

### Statistical analysis

Propensity score matching (PSM) was conducted in R to address selection bias in this observational study by pairing patients with similar controls, effectively controlling for confounders including NCCN risk group, Gleason grade, TNM stage, age, and PSA. R4.3.1 software performed 1:4 nearest neighbor matching, resulting in 12*4 matched samples. Kaplan-Meier analysis in SPSS v26 assessed survival differences, with log-rank tests comparing treatment groups. Chi-square and Student’s t-test detected differences in categorical and continuous variables. All tests were two-sided, with significance set at P<0.05.

## Results

### Basic parameters

The study involved 12 high-dose and 48 control patients. Specifically, in the high-dose group, there was 1 patient with low risk, 2 with unfavorable intermediate risk, 6 with high risk, and 3 with very high risk. In the control group, there were 4 patients with low risk, 12 with unfavorable intermediate risk, 22 with high risk, and 10 with very high risk. Last follow-up was May 2023 or death. The median follow-up period extended to 74.0 months (range 5.3-117.0 months). The high-dose group had 75% high/very high-risk patients, with 33% undergoing hormonal therapy. Four patients succumbed to prostate cancer progression, while five had non-cancer-related deaths, including three strokes and two pneumonia cases. Among these non-cancer-related deaths, one patient had an unfavorable intermediate-risk (NCCN classification), and four were high-risk. Of the four patients who died due to prostate cancer metastasis, one had multiple advanced metastases affecting the lungs and bones. Another patient exhibited metastases in various locations, including the lumbar spine and multiple bones. In the remaining two cases, systemic metastatic progression was considered. A total of 47 patients (78.3%) had pre-existing health conditions, such as diabetes, hypertension, and coronary heart disease. Specific patient demographics are detailed in **Table 2**.

**Table 2:** Patients demography and tumor characteristics.

Characteristics	Total	The high-dose group	The control group
Total patients, n	60	12	48
Follow-up, median (SD)	74.0 (5.3-117.0)	52.8 (14.5-99.3)	76.9 (5.3-117.0)
Age at treatment time-years, median (SD)	73.5 (54-83)	72 (65-78)	74 (54-83)
PSA, median (SD)	17.1 (0.4-100)	17.1 (0.4-100)	18.4 (0.4-91)
Gleason score			
• 3 + 3 = 6	13	1	12
• 3 + 4 = 7,4 + 3 = 7	23	5	18
• 4 + 4 = 8,3 + 5 = 8,5 + 3 = 8	14	3	11
• 5 + 4 = 9,4 + 5 = 9	10	3	7
Clinical T-stage			
• T_2a_	25	5	20
• T_2b_	5	1	4
• T_2c_	23	4	19
• T_3a_	2	1	1
• T_3b_	5	1	4
NCCN risk grouping			
• Low	3	1	2
• Unfavorable intermediate	16	2	14
• High	30	6	24
• Very high	11	3	8
ECOG score			
• 0	2	1	1
• 1	58	11	47
Pre-treatment TURP			
• Yes	14	6	8
• No	46	6	40
Synchronize/previously used ADT			
• No	45	5	40
• Yes	15	7	8
Pre-existing health conditions			
• No	13	6	7
• Yes	47	6	41

### Survival differences

**Figure 1:**
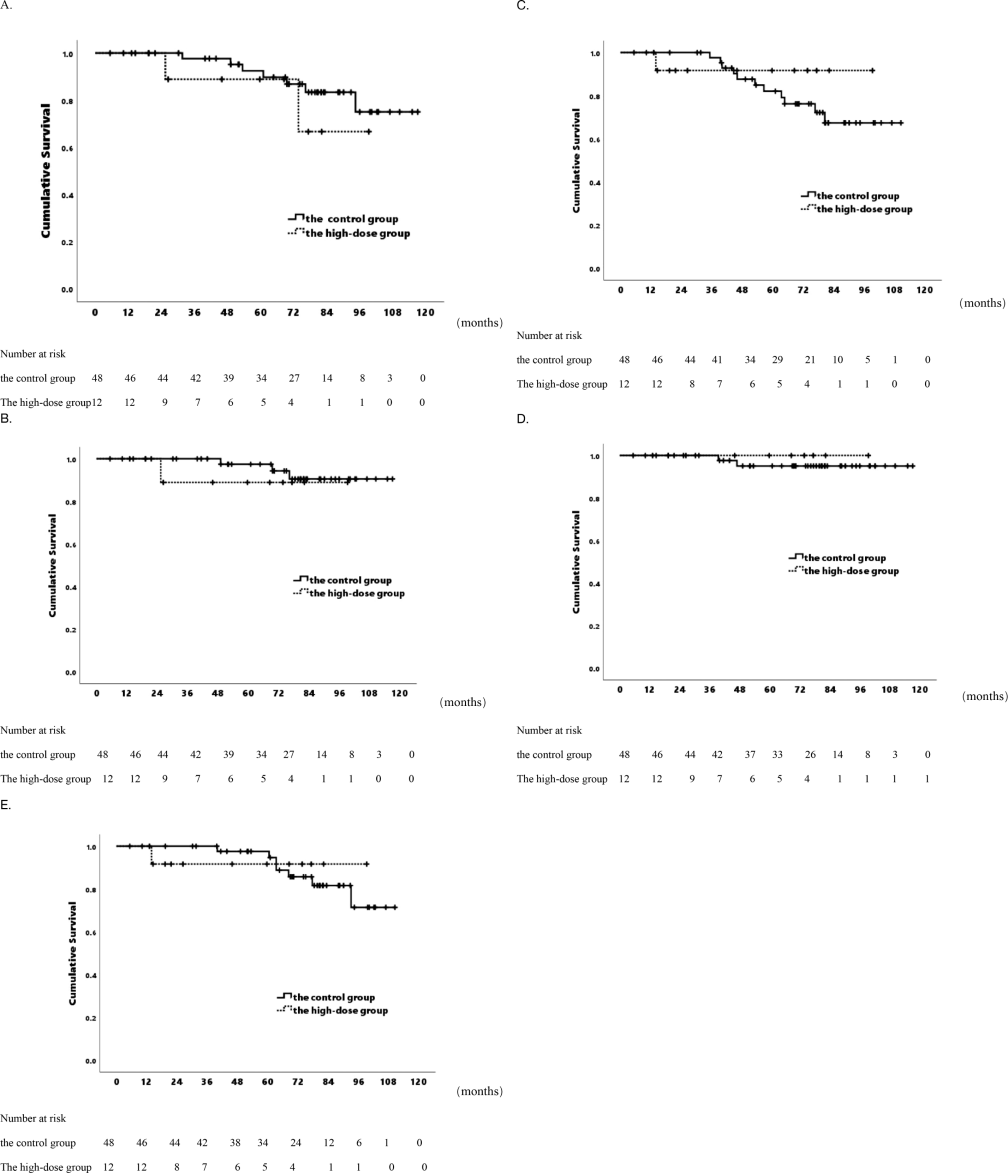
Actuarial survival analysis of patients in two distinct groups. **(A)** Overall survival. **(B)** Cancer-specific Survival. **(C)** Biochemical Progression-free Survival. **(D)** Local Control Survival. **(E)** Distant Metastasis-free Survival.

For 60 patients, 5-year OS was 91.7%, 7-year OS was 81%, median OS was 104.1 months (range 96.5-111.7). In the high-dose group, 5-year OS was 88.9%, 7-year OS was 66.7%, respectively. In the control group, the corresponding rates were 92.5% and 83.4% (p=0.402& **Figure 1A**). CSS rates at 5 and 7 years were 95.9% and 89.9%, median CSS 111.2 months (range 105.6-116.7). For CSS, the high-dose group had a 5-year rate of 88.9%, a 7-year rate of 88.9%, while the control group had a 5-year rate of 97.4%, a 7-year rate of 90.5% (p=0.480& **Figure 1B**). Dosimetric data related to BED were analyzed, and the correlation between clinical/patient baseline information and OS as well as prostate CSS was examined. Univariate analysis showed no significant correlation between prostate CSS or OS and clinical characteristics, patient baseline information, or SBRT parameters (**Supplementary Tables S1**, **S2**). Despite increased radiation dosage for a higher BED, there was no improvement in patient survival.

### bPFS differences

In the 60-patient cohort, 5- and 7-year bPFS rates were 83.2% and 70.2%, with a median bPFS of 94.1 months (range 86.1-102.2 months). The high-dose group exhibited a 5-year bPFS of 91.7%, and 7-year bPFS of 91.7%, while the control group had a 5-year bPFS of 82.1%, 7-year bPFS of 67.4% (p=0.497& **Figure 1C**). No differences in biochemical control were observed. Univariate analysis revealed an association between elevated Gleason score and impaired biochemical control. Gleason score > 7 showed 5-year bPFS of 72.4%, 7-year bPFS of 55.5%, compared to Gleason score ≦ 7 with 5-year bPFS of 95.8%, 7-year bPFS of 89.8% (p=0.028). Additionally, Patients with more adverse classifications had suboptimal biochemical control (p=0.028) (**Table 3**). In the low/unfavorable intermediate risk group, 100% bPFS was achieved at both 5- and 7-year, while in the high/very high-risk group, 5-year bPFS was 77.4%, and 7-year bPFS was 60.6%.

**Table 3:** Univariate analysis for bPFS rate.

Factors	5-year rate (%)	7-year rate (%)	P value
Dose group			0.497
The high-dose group	91.7	91.7	
The control group	82.1	67.4	
Gleason Score			0.028
≦7	95.8	89.8	
> 7	72.4	55.5	
PSA at diagnosis-ng/ml			0.128
≦20	93.1	86.0	
>20	72.4	61.2	
NCCN risk grouping			0.028
Low+Unfavorable intermediate	100	100	
High+very high	77.4	60.6	
Treatment regimen:			0.520
Daily treatment	88.9	88.9	
Alternate-day treatment	82.1	67.4	
Age-years			0.846
<70	76.5	76.5	
≧70	86.9	68.6	
Synchronize/previously used ADT			0.833
Presence	92.3	65.6	
Absence	80.6	72.1	
TURP before SBRT			0.626
Yes	93.3	81.7	
No	80.6	67.6	

### LC and DMFS differences

Over 5- and 7-year periods, the cohort exhibited consistent LC rates of 95.7% and 95.7%, with an average follow-up of 113.9 months (range 109.6-118.1 months). The high-dose group achieved a 7-year LC rate of 100%, while the control group had a 7-year rate of 95.1% (p=0.569& **Figure 1D**). Additionally, DMFS rates at 5- and 7-year were 96.2% and 82.3%, averaging 99.8 months(range 93.1-106.6 months). The high-dose group had 5-year and 7-year DMFS rates of 91.7%, while the control group had rates of 97.6% and 81.6%, respectively (p=0.918& **Figure 1E**). In the patient cohorts, no significant correlation was observed between clinical patient information or SBRT parameters and LC (**Supplementary Table S3**). Univariate analysis revealed that patients with pre-existing health conditions such as hypertension, heart disease, and diabetes, were less prone to distant metastasis than those without (p=0.047). Patients with pre-existing conditions: 5-year DMFS 97.4%, 7-year DMFS 90.8%. Patients without such conditions: 5-year DMFS 92.3%, 7-year DMFS 46.9%. A higher distant metastasis trend was noted in Gleason >7 vs. ≤7 (p=0.060). No significant correlations with other clinical or treatment factors (**Supplementary Table S4**).

### Overall toxicity

The majority of patients showed good tolerance to SBRT, with no grade 3+ adverse reactions observed. The high-dose group had no significant toxicity. In the control group (48 patients), one (2.1%) had grade 2 acute genitourinary (GU) toxicity, while two (4.2%) reported grade 1 acute GU symptoms. Two (2.3%) had grade 2 late GU toxicity. No acute or late gastrointestinal (GI) toxicity occurred. Common treatment-related adverse effects: hematuria, urinary frequency, nocturia, urinary pain, and difficulty in urination. All acute toxicities were transient, reversible with medication, and didn’t hinder treatment completion.

## Discussion

In this retrospective study, PSM matching addressed efficacy bias by balancing high-dose and control groups. Results showed no significant differences in OS, CSS, bPFS, LC, or DMFS between them. The study supports NCCN’s recommendation of 36.25 Gy in five fractions for localized PCa patients undergoing SBRT, achieving a BED below 250 Gy.

SBRT, recommended for localized PCa, involves ultra-hypofractionated radiotherapy (7–10 Gy per fraction over 4–5 fractions) with a BED up to EQD_2–_ 164 Gy, given within 1–2 weeks. It’s considered the standard of low- and intermediate-risk PCa, demonstrating excellent oncological outcomes. The HYPO-RT-PC trial in Scandinavia, compared ultra-hypofractionation (7 fractions of 42.7 Gy) with traditional fractionation (39 fractions of 78.0 Gy) (4). This comparison used 3D conformal radiation therapy (3DCRT), IMRT, or volumetric modulated arc therapy (VMAT) for intermediate or high-risk PCa. Ultra-fractionation proved non-inferior, with no difference in cancer-specific mortality or OS. Though associated with acute GU and GI symptoms, no difference was seen in late symptoms or overall QoL.

The PACE-B study (874 patients, low and favorable intermediate-risk PCa) compared conventional (78 Gy in 39 fractions over 8 weeks), moderately hypofractionated radiotherapy (62 Gy in 20 fractions over 4 weeks), and SBRT (36.25 Gy in 5 fractions). Results showed that SBRT’s shorter duration didn’t increase acute toxicity (10). The American Society for Radiation Oncology (ASTRO), the American Society of Clinical Oncology (ASCO), and the NCCN guidelines consider prostate SBRT acceptable for localized PCa (11). A meta-analysis (38 studies, 6,116 patients) on prostate cancer patients undergoing SBRT for low-, intermediate-, and high-risk diseases with fewer than 10 fractions exceeding 5 Gy demonstrated 5-year and 7-year biochemical recurrence-free survival rates (BRFS) rates of 95.3% and 93.7%. Estimated late grade 3 or higher GU and GI toxicity rates were 2.0% and 1.1%, respectively. This evidence supports SBRT as a standard for localized PCa. The review had a 39-month median follow-up, incorporating the HYPO-RT-PC trial. 80% of ultra-hypofractionation cases used 3D-CRT, with the rest using IMRT/VMAT (14). In a Phase II trial, localized SBRT (36.25 Gy/5 every other day) yielded promising outcomes. A 36-month follow-up showed a 96% 3-year bPFS rate, with all 24 eligible patients avoiding salvage prostatectomy. No grade 3 or higher toxicities were observed, indicating minimal impact on patients’ QoL (15). Another Phase II trial, HYPOSTA, explored hypofractionated robotic SBRT on 85 localized PCa patients. Using the CyberKnife system (35 Gy/5f), it showed favorable short-term toxicity profiles, especially for intermediate or high-risk cases involving the proximal seminal vesicles (16).

In conventional radiotherapy, NRG 0126 compared 70 Gy to a dose-escalated 79.2 Gy at 1.8 Gy per fraction in a similar group of intermediate-risk patients (n=1532). The report indicated that dose-escalated treatment, relatively better tolerated, significantly reduced distant metastasis (17). Therefore, the benefit of reducing distant metastasis with dose escalation in a larger SBRT patient cohort, as seen in RTOG 0126, might translate into a modest yet statistically significant improvement in OS (18). Previous studies also reported that dose-escalated SBRT can enhance BRFS rates compared to lower-dose SBRT in low- and intermediate-risk prostate cancer. However, these studies failed to detect potential improvements in OS or DMFS rates, alternative endpoints for prostate cancer patients (19). Other studies utilized different dose regimens. Meier et al. studied 309 low/intermediate-risk prostate cancer patients with robotic SBRT (40 Gy/5f for prostate, 36.25 Gy/5f for seminal vesicles), reporting minimal toxicity and 95.6% 5-year OS (20). Additionally, HYPO-RT-PC trial showed non-inferiority for freedom from failure (FFS) with 42.7 Gy/7f vs. conventional 78.0 Gy/39f in intermediate- and high-risk patients (4).

Boike et al. conducted a phase I dose-escalation study for low-risk and intermediate-risk prostate cancer treated with SBRT. In a prospective cohort of 15 patients, dose escalation ranged from 45 Gy to 50 Gy, administered in fractions of 9, 9.5, and 10 Gy each, every other day, using a rectal balloon for protection. The study reported 18% grade 2 and 2% grade 3 rectal toxicity, and 31% grade 2 and 4% grade 3 GI toxicity. Importantly, dose escalation to 50 Gy was completed without dose-limiting toxicity (21). Another study examined 24 patients with intermediate- and high-risk prostate cancer undergoing dose-escalated prostate and proximal seminal vesicle SBRT. High-dose avoidance zones(HDAZ) were established, and patients achieved a 24-month PSA recurrence-free survival of 95.8% (22). In a dose-escalation study involving 75 patients with low- or intermediate-risk localized PCa, three SBRT dose levels were explored: 35 Gy, 37.5 Gy, and 40 Gy in 5 fractions. The 2-year incidence rates of Grade 2 late GU and GI toxicities were 34% and 7%, respectively, with higher doses associated with increased GU toxicity. No Grade 3 GI or Grade 4 acute GU toxicities or Grade 3 late toxicities were observed. Prescription of 35 Gy/5f was less likely to cause adverse events, suggesting caution with higher SBRT doses (23). In a retrospective study of 2214 intermediate-risk prostate cancer patients treated with SBRT, a dose of 36.25 Gy/5f was compared to 35 Gy/5f. Despite a small dose difference, the increase in BED from 35 Gy/5f to 36.25 Gy/5f was associated with improved survival (24). Our study explored the survival outcomes of higher BED in SBRT for localized prostate cancer. Although the high-dose group showed better trends in bPFS and LC rates, no statistically significant improvements in tumor control or survival were found. These results align with NCCN guidelines recommending a dosage of 36.25 Gy to 35 Gy in 5 fractions for localized prostate cancer SBRT. Furthermore research is needed on dose-response relationships. Furthermore, with the advancement of artificial intelligence and big data technologies, leveraging multidimensional dosimetric parameters for precise modeling and risk prediction will provide robust support for personalized radiotherapy dose optimization.

Our cohort, despite reporting fewer toxicities, demonstrated potential SBRT related toxicity compared to moderately fractionated IMRT. A retrospective study (n=4,005) reported higher GI toxicity with SBRT than IMRT at 24 months (44% vs. 36%; P = 0.001) (25). Prospective evaluation by K. et al. with 205 patients undergoing SBRT treatment (37.5–40 Gy/5f) using the “CyberKnife M6” showed mild to moderate early side effects, with GU and GI acute radiation-related side effect rates reported as GU: grade 0 - 17.1%, grade 1 - 30.7%, grade 2 - 50.7%, grade 3 - 1.5%; GI: grade 0 - 62.4%, grade 1 - 31.7%, grade 2 - 5.9%, grade 3 - 0%, and no grade 4 or higher toxicities (26). MRI-guided SBRT in prostate cancer radiation therapy demonstrated favorable outcomes, with the use of a 1.5-Tesla MR linear accelerator showing feasibility and safety. Comparative analysis suggests MR-guided Radiation Therapy (MRgRT) may reduce overall Grade 1 acute toxicity at six months, with a declining trend in Grade 2 GI toxicity (27). The MIRAGE trial indicated MRI-guided SBRT significantly decreased physician-assessed moderate acute toxicity and patient-reported declines compared to CT guidance (28). Integrating SBRT with prophylactic pelvic radiation, along with gross tumor volume within the prostate (GTVp) augmentation guided by multiparametric magnetic resonance imaging (mpMRI), proved effective and well-tolerated for high-risk PCa patients (29). Future studies could build on conventional prostate radiation doses by incorporating advanced imaging techniques such as PSMA PET/MR to enable targeted dose escalation to active lesions, aiming to further reduce toxicity and improve local control. Moreover, personalizing SBRT dose strategies will likely depend on integrating molecular biomarkers that reflect tumor-specific stress responses, immune activity, and metabolic pathways. Cross-cancer insights—such as CISD2-mediated iron homeostasis in HNSCC, GLS-driven glutamine metabolism in breast cancer, NT5E-associated purine signaling in pancreatic tumors, and efferocytosis-related immune evasion in glioblastoma—underscore how microenvironmental dysregulation can drive radioresistance and recurrence (30–33). Applying such multi-omic biomarker frameworks to prostate cancer holds promise for identifying patients who are more suitable for dose escalation, while allowing deintensification in low-risk cases. This approach could optimize therapeutic indices and advance truly risk-adapted SBRT.

This study had limitations. It was retrospective, introducing bias and limiting causal inferences. The small sample size (12 high-dose, 48 control) indicated limited statistical power and heterogeneity. Despite 1:4 PSM controlling for measured variables, unmeasured confounders could influence treatment decisions. Furthermore, in comparison to other studies, the study didn’t rule out potential differences related to ethnicity, specifically between East Asian and Western populations. Dose prescriptions (9.5*4, 7.25-8*5, and 6.1*7) align with NCCN guidelines, but escalation beyond STAMPEDE trial equivalents wasn’t recommended due to known toxicity increase without improved OS. In our analysis, higher SBRT doses didn’t correlate with enhanced survival outcomes or significantly different toxicities.

## Conclusion

In SBRT treatment for localized PCa, while the high-dose group showed an upward trend in BPFS and LC rates at 5 and 7 years compared to the control group, the adoption of a high biologically effective dose did not significantly improve tumor control rates and survival. Clinicians should weigh treatment effectiveness and potential adverse effects when devising personalized treatment plans to maximize therapeutic benefits for patients.

## Data Availability

The raw data supporting the conclusions of this article will be made available by the authors, without undue reservation.

## Glossary

BED: biologically effective dose
SBRT: Stereotactic Body Radiotherapy
PCa: prostate cancer
PSM: propensity score matching
PSA: prostate-specific antigen
NCCN: National Comprehensive Cancer Network
OS: overall survival
CSS: cancer-specific survival
bPFS: biochemical progression-free survival
LC: local control
DMFS: distant metastasis-free survival
AS: active surveillance
RP: radical prostatectomy
EBRT: external beam radiotherapy
FT: focal therapy
QoL: quality of life
IMRT: intensity-modulated radiotherapy
RBE: relative biological effectiveness
MRI: magnetic resonance imaging
ECT: emission computed tomography
ECOG: Eastern Cooperative Oncology Group
ADT: androgen deprivation therapy
CT: computed tomography
CTV: clinical target volume
PTV: planning target volume
OAR: organs at risk
CTCAE: Common Terminology Criteria for Adverse Events
GU: genitourinary
GI: gastrointestinal
EQD_2_: equivalent dose in 2 Gy per fraction
3DCRT: 3D conformal radiation therapy
VMAT: volumetric modulated arc therapy
ASTRO: American Society for Radiation Oncology
ASCO: American Society of Clinical Oncology
BRFS: biochemical recurrence-free survival rates
FFS: freedom from failure
HDAZ: high-dose avoidance zones
MRgRT: MR-guided radiation therapy
GTVp: gross tumor volume within the prostate
mpMRI: multiparametric magnetic resonance imaging
