# Supplementary material for: Analysis of standard vs dose-escalated stereotactic body radiation therapy in localized prostate cancer: a comparative evaluation of survival outcomes
Source: Front Immunol. 2025 Aug 13;16:1654174. doi: 10.3389/fimmu.2025.1654174 (PMC12380758; doi:10.3389/fimmu.2025.1654174)
Supplement: Supplementary file 1 [file DataSheet1.docx]

**FIGURE S1 | Distribution graph of BED_1.5_ (Gy) in two distinct patient groups.**


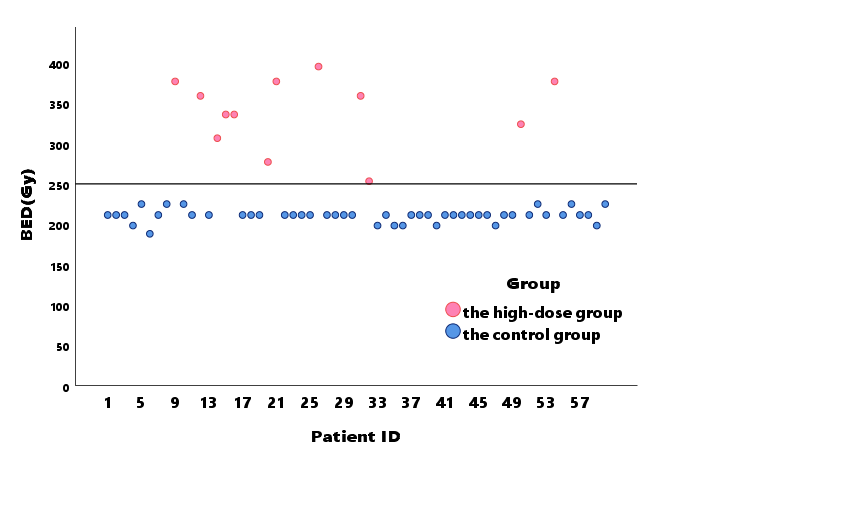


**Table S1. Univariate analysis for OS rate.**

| Factors | 5-year rate (%) | 7-year rate (%) | P value |
| --- | --- | --- | --- |
| Dose group |  |  | 0.402 |
| The high-dose group | 88.9 | 66.7 |  |
| The control group | 92.5 | 83.4 |  |
| Gleason Score |  |  | 0.467 |
| ≦7 | 90.9 | 79.5 |  |
| > 7 | 92.3 | 82.8 |  |
| PSA at diagnosis-ng/ml |  |  | 0.260 |
| ≦20 | 93.1 | 87.6 |  |
| >20 | 90.4 | 74.0 |  |
| NCCN grouping |  |  | 0.339 |
| Low+Unfavorable intermediate | 93.3 | 93.3 |  |
| High+very high | 91.4 | 76.8 |  |
| Treatment regimen |  |  | 0.402 |
| Daily treatment | 88.9 | 66.7 |  |
| Alternate-day treatment | 92.5 | 83.4 |  |
| Age-years |  |  | 0.375 |
| <70 | 83.1 | 71.2 |  |
| ≧70 | 96.4 | 85.0 |  |
| Presence of BPH |  |  | 0.897 |
| Presence | 91.2 | 87.3 |  |
| Absence | 93.3 | 79.4 |  |
| TURP before SBRT |  |  | 0.106 |
| Yes | 91.7 | 69.8 |  |
| No | 91.8 | 84.5 |  |
| Associated symptoms |  |  | 0.101 |
| Presented | 81.2 | 74.4 |  |
| None | 97.1 | 85.0 |  |

**OS: Overall survival; TURP: Transurethral resection of the prostate; SBRT: Stereotactic body radiation therapy**; **BPH :Benign prostatic hyperplasia**

**Table S2.** **Univariate analysis for Prostate cancer-specific OS rate.**

| Factors | 5-year rate (%) | 7-year rate (%) | P value |
| --- | --- | --- | --- |
| Dose group |  |  | 0.480 |
| The high-dose group | 88.9 | 88.9 |  |
| The control group | 97.4 | 90.5 |  |
| Gleason Score |  |  | 0.468 |
| ≦7 | 100 | 92.9 |  |
| > 7 | 92.3 | 87.2 |  |
| PSA at diagnosis-ng/ml |  |  | 0.248 |
| ≦20 | 96.7 | 96.7 |  |
| >20 | 95.5 | 82.7 |  |
| NCCN risk grouping |  |  | 0.212 |
| Low+Unfavorable intermediate | 100 | 100 |  |
| High+very high | 94.4 | 86.1 |  |
| Treatment regimen: |  |  | 0.480 |
| Daily treatment | 88.9 | 88.9 |  |
| Alternate-day treatment | 97.4 | 90.5 |  |
| Age-years |  |  | 0.550 |
| <70 | 100.0 | 95.8 |  |
| ≧70 | 88.0 | 75.4 |  |
| Synchronize/previously used ADT |  |  | 0.874 |
| Presence | 91.7 | 91.7 |  |
| Absence | 97.2 | 89.4 |  |
| TURP before SBRT |  |  | 0.845 |
| Yes | 91.7 | 91.7 |  |
| No | 97.2 | 89.5 |  |
| Associated symptoms |  |  | 0.364 |
| Presented | 88 | 88.0 |  |
| Absence | 100 | 91.6 |  |

**OS: Overall survival; TURP: Transurethral resection of the prostate; SBRT: Stereotactic body radiation therapy; ADT: Androgen deprivation therapy**

**Table S3. Univariate analysis for LC rate.**

| Factors | 5-year rate (%) | 7-year rate (%) | P value |
| --- | --- | --- | --- |
| Dose group |  |  | 0.569 |
| The high-dose group | 100 | 100 |  |
| The control group | 95.1 | 95.1 |  |
| Gleason Score |  |  | 0.171 |
| ≦7 | 100 | 100 |  |
| > 7 | 91.8 | 91.8 |  |
| PSA at diagnosis-ng/ml |  |  | 0.136 |
| ≦20 | 100 | 100 |  |
| >20 | 91.1 | 91.1 |  |
| NCCN risk grouping |  |  | 0.392 |
| Low+Unfavorable intermediate | 100 | 100 |  |
| High+very high | 94.2 | 94.2 |  |
| Treatment regimen: |  |  | 0.569 |
| Daily treatment | 100 | 100 |  |
| Alternate-day treatment | 95.1 | 95.1 |  |
| Age-years |  |  | 0.588 |
| ﹤70 | 94.1 | 94.1 |  |
| ≧70 | 96.8 | 96.8 |  |
| Synchronize/previously used ADT |  |  | 0.474 |
| Presence | 100 | 100 |  |
| Absence | 94.7 | 94.7 |  |
| TURP before SBRT |  |  | 0.474 |
| Yes | 100 | 100 |  |
| No | 94.7 | 94.7 |  |
| Associated symptoms |  |  | 0.563 |
| Presented | 93.8 | 93.8 |  |
| Absence | 96.8 | 96.8 |  |
| Hematuria or infection |  |  | 0.597 |
| Presented | 100 | 100 |  |
| Absence | 95.1 | 95.1 |  |

**LC: local control; TURP: Transurethral resection of the prostate; SBRT: Stereotactic body radiation therapy; ADT: Androgen deprivation therapy**

**Table S4. Univariate analysis for DMFS rate.**

| Factors | 5-year rate (%) | 7-year rate (%) | P value |
| --- | --- | --- | --- |
| Dose group |  |  | 0.918 |
| The high-dose group | 91.7 | 91.7 |  |
| The control group | 97.6 | 81.6 |  |
| Gleason Score |  |  | 0.060 |
| ≦7 | 100 | 93.8 |  |
| > 7 | 92.7 | 74.3 |  |
| PSA at diagnosis-ng/ml |  |  | 0.389 |
| ≦20 | 96.9 | 88.8 |  |
| >20 | 95.7 | 73.4 |  |
| NCCN risk grouping |  |  | 0.102 |
| Low+Unfavorable intermediate | 100 | 100 |  |
| High+very high | 94.7 | 76.4 |  |
| Treatment regimen: |  |  | 0.960 |
| Daily treatment | 88.9 | 88.9 |  |
| Alternate-day treatment | 97.6 | 81.6 |  |
| Age-years |  |  | 0.303 |
| <70 | 89.1 | 82.2 |  |
| ≧70 | 100 | 83.0 |  |
| Synchronize/previously used ADT |  |  | 0.258 |
| Presence | 92.3 | 61.5 |  |
| Absence | 97.4 | 97.4 |  |
| TURP before SBRT |  |  | 0.872 |
| Yes | 93.3 | 81.7 |  |
| No | 97.4 | 82.8 |  |
| Associated symptoms |  |  | 0.725 |
| Presented | 89.5 | 81.4 |  |
| Absence | 100 | 83.7 |  |
| Hematuria or infection |  |  | 0.265 |
| Presented | 100 | 100 |  |
| Absence | 95.5 | 79.9 |  |
| Inherent medical comorbidities |  |  | 0.047 |
| Presented | 97.4 | 90.8 |  |
| Absence | 92.3 | 46.9 |  |

**DMFS: Distant metastasis-free survival; TURP: Transurethral resection of the prostate; SBRT: Stereotactic body radiation therapy; ADT: Androgen deprivation therapy**
